# Perspective: Call for Re-evaluation of the Tolerable Upper Intake Level for Magnesium Supplementation in Adults

**DOI:** 10.1016/j.advnut.2023.06.008

**Published:** 2023-07-22

**Authors:** Rebecca Costello, Andrea Rosanoff, Forrest Nielsen, Christina West

**Affiliations:** CMER, Center for Magnesium Education and Research, Pahoa, Hawaii, United States

**Keywords:** magnesium, diarrhea, adverse events, tolerable upper intake level, supplements

## Abstract

In 1997, the US Institute of Medicine (IOM) dietary reference intakes (DRI) Committee established a magnesium (Mg) tolerable upper intake level (UL) for adults of 350 mg/d from supplemental intake alone. Diarrhea was the limiting factor. The safety of oral Mg dietary supplements exceeding the UL is currently in debate. Increasing the UL may result in more Mg supplementation, decreasing the prevalence of undernutrition for this nutrient and thus providing additional protection against numerous chronic diseases. This perspective aims to show that more recent and comprehensive evidence-based data on the occurrence of diarrhea indicate that the Mg UL for adults should be re-evaluated. To update the literature base to re-evaluate setting the Mg UL, a PubMed search was conducted to identify intervention studies published between 1997 and 2022 that used single-ingredient Mg products reporting a priori diarrhea adverse events among adults. The Food and Drug Administration Center for Food Safety and Adverse Event Reporting System (CAERS) was also searched for adverse events caused by Mg supplementation. The PubMed search identified 10 studies, including 5 meta-analyses and 5 randomized controlled trials, that met the search criteria. Seven studies (Mg intakes of 128–1200 mg/d) found no significant differences in diarrhea occurrence between the intervention and control groups. One meta-analysis found only minor differences in gastrointestinal disturbances between groups given placebo versus 520 mg Mg/d, but withdrawals were not significantly different between groups. Another meta-analysis found that 3 of 13 studies (120–973 mg/d) reported diarrhea that led to study withdrawal, but the treatment arm was not specified in 2 studies. The CAERS search, when limited to single-ingredient *suspect* Mg products, found only 40 attributable cases of gastrointestinal adverse events. Only one-third of these 40 cases noted a complaint of diarrhea. These updated data indicate that doses above the current UL for Mg supplements can be consumed without adverse events.

**Statement of Significance**

Data suggest that increasing the tolerable upper intake level for magnesium supplements is safe and may decrease the prevalence of individuals not meeting their need for this nutrient, which contributes to protection against numerous chronic diseases.

## Introduction

In setting a tolerable upper intake level (UL) for magnesium (Mg) in 1997, the US Institute of Medicine (IOM) dietary reference intakes (DRI) Committee did not find any reports of adverse effects attributable to high intakes of naturally occurring Mg in foods [1]. However, the committee noted that 4 studies reported pharmacological or high supplemental doses (>350 mg/d) of Mg salts causing gastrointestinal disturbances such as abdominal cramping and diarrhea. Thus, instead of setting a UL based on total intake of Mg from all sources, as has been done for all other essential nutrients, including vitamin C, with similar effects with supplemental use, the DRI Committee established a UL for Mg from supplemental sources only. The Mg UL was set at 350 mg/d because “some individuals in the population may be at risk of a mild, reversible adverse effect (diarrhea) even at doses from nonfood sources that are easily tolerated by others” [1]. The committee also found that in 1986, approximately 5% of men and >5% of women who used Mg supplements exceeded the UL of 350 mg/d [2]. Yet in a search of adverse events reported since the inception of the FDA Center for Food Safety and Applied Nutrition Adverse Event Reporting System (CAERS) in January 2004 until June 30, 2022, only 40 cases of gastrointestinal adverse effects were attributed to single-ingredient *suspect* Mg products and only one-third of these noted a complaint of diarrhea [3]. This low incidence indicates that the UL for Mg and the basis for which it was established should be re-evaluated.

A re-evaluation of the UL for Mg also is predicated by it currently being at a level that may conflict with an amount close to that needed by some individuals to meet their total requirement to prevent a chronic latent Mg deficit. A chronic latent Mg deficit puts the individual at risk for developing hypertension [4,5], cardiovascular disease [4,6], and diabetes-related metabolic risk factors [4,7] even though their daily Mg intake falls within published recommended requirements and their serum Mg level is within the currently recommended “normal” range.

Although a diet incorporating Mg-rich foods, including unrefined grains, legumes or beans, nuts, seeds, and green vegetables, is recommended to prevent Mg deficiency, this recommendation apparently is not well adopted in the United States. It has been reported that over half of the US population aged ≥20 y consumes less than the current estimated average requirement (EAR) of Mg of 330–350 mg/d for adult men, 300–335 mg/d for adult women, and 330–335 mg/d for pregnant women depending on age [8]. The number of individuals not meeting these requirements might be even higher because current EARs, RDAs, and DRIs are based on poorly controlled, unreliable balance data in the United States [9] and United Kingdom [10] and on usual dietary intakes established by the World Health Organization [11] and the European Food Safety Authority [12]. Recent well-controlled balance data have indicated that these DRIs need to be re-evaluated and must consider the increasing need for Mg with increasing body weights [4,13,14], high calcium intakes [15], and the impact of long-term medication use [16]. The evidence for the widespread inadequate intake of Mg supports the conclusion of the 2015–2020 Dietary Guidelines for Americans that Mg is an under-consumed nutrient [9]. Supplements high in Mg may be needed to meet requirements.

An update of the UL for Mg using data from studies with improved measures of adverse events reported since 1997 is needed to ensure that individuals concerned about adequate nutritional intakes are aware of the best estimates for safe intakes for this nutrient. In a study of 6 NHANES cycles (between 1999-2000 and 2009-2010) with 30,899 adults aged ≥20 y, 24,763 participants reported using dietary supplements in the past 30 d [17,18]; 33.3% used Mg supplements (mean, 146.8 mg/d). Although the number of adults with intakes below the EAR decreased as a result of Mg supplementation, 49.7% of those surveyed still had total nutrient intakes below the requirement.

A recent economic report [19] suggested that the calculated relative risk reduction of a coronary artery disease-related event by individuals using Mg supplements at preventive intake levels of 400 mg/d was 5.34%. This finding was based on data from a meta-analysis (6) of prospective cohort studies after controlling for variability due to sample size, research methodologies and study protocols, and patient population differences. The expected reduction in expenditures, or cost savings in 2022 from avoided events due to coronary artery disease, would have been $3.01 billion.

This perspective summarizes the past data used to set the current UL for Mg, along with some of their shortcomings. The most recent and more comprehensive evidence-based data are also presented, which could be used for the proposed re-evaluation of the UL for Mg from dietary supplements if that is the benchmark instead of total Mg intake for the UL of this nutrient. The efficacy of Mg supplementation is outside the scope of this work and is not addressed here.

## Setting and shortcomings of the 1997 UL for Mg

Table 1 describes the 10 studies [[20], [21], [22], [23], [24], [25], [26], [27], [28], [29]] used by the DRI Committee in setting the UL for Mg in 1997 [1]. Because no adverse effects caused by food intake were found, a UL of 350 mg (14.6 mmol)/d as an Mg supplement for adolescents and adults was established based on a lowest observed adverse event level (LOAEL) of 360 mg (15 mmol)/d and an uncertainty factor of 1.0. Six of the studies reviewed in setting the UL documented no gastrointestinal symptoms or complaints [20,[24], [25], [26],^28^,^29^], and 2 studies [20,24] did not comment on gastrointestinal complaints. Thus, the UL was based on findings from the following 4 studies, all of which may have shortcomings with their use.

**TABLE 1:** Studies used by the Institute of Medicine to determine the 1997 tolerable upper intake level for Mg^a^

Reference	Population	Sample size	Study design	Mg formulation	Daily Mg dose	Duration	Side effects/AEs	Comments
Altura et al., 1994 (20)	Healthy men (aged 18–38 y)	18, with 40 age-matched volunteers as controls	Triple crossover RCT	Mg saturation period, followed by 3 diets enriched with Mg (phosphate and oxide, 200 mg); 250 mg MgO in smooth gelatin capsule, and 250 mg MgO in hard gelatin capsule	All equivalent to 300 mg (12.34 mmol)	6 d	No comment on GI symptoms	Total Mg (food, Mg-rich water, and supplement) intake during the saturation period totaled 1181–1531 mg (48.6–63 mmol); on test days, it totaled 652 mg (16.3 mmol)
Bashir et al., 1993 (21)	Men and women (aged 51–70 y)	21 (19 completed)	Crossover	MgCl	360 mg (15.8 mmol)	6 wk	GI; 2 patients reported diarrhea	For patients with stable CHF taking diuretics, mean ejection fraction of 25.9%
Fine et al., 1991 (22) (a priori)	Healthy adults (aged 25–35 y)	19 (18 men and 1 woman)	Metabolic study	Mg(OH)_2_	1166, 2333, and 4690 mg/d (48, 96, and 193 mmol/d)	4 d, 11 collection periods	Authors determined that Mg contributed to 21 cases of diarrhea among 359 individuals with chronic diarrhea	To study the diarrhea threshold by induced osmotic diarrhea and monitor fecal Mg losses
Marken et al., 1989 (23)	Healthy Black and White men and women (aged 21–50 y)	50 (41 completed)	Double-blind crossover RCT	MgO	476 mg (19.6 mmol)	60 d	18 developed diarrhea; 6 dropouts due to diarrhea (5 receiving Mg, 1 receiving placebo)	Higher-than-expected rate of diarrhea; some symptoms were alleviated by taking Mg with food
Nadler et al., 1992 (24)	Adults with type 2 diabetes (aged 26–65 y)	20 (12 men and 8 women)	Metabolic ward study	MgO or MgCl	400 mg (16.7 mmol)	8 wk	2 patients with mild diarrhea; resolved spontaneously	16 patients had diabetes that was controlled with insulin, and 4 were taking oral hypoglycemic agents
Nagy et al., 1988 (25)	Adult men and women with acute duodenal ulcer (mean age, 34.4 y)	20	RCT	Aluminum-Mg-hydroxycarbonate antacid (TISACID)	≤1200 mg (50 mmol)	6 wk	None of the 20 patients had diarrhea	Clinical pharmacological tolerability study. Cimetidine was administered with study intervention
Paolisso et al., 1992 (26)	Nonobese healthy elderly (mean age, 77.8 ± 2.1 y) and young healthy (mean age, 36.1 ± 0.4 y) individuals	25 young individuals and 12 elderly individuals	Double-blind crossover RCT	Mg pidolate	395 mg (16.2 mmol)	4 wk	No comment on GI complaints	Elderly individuals were insulin resistant compared with young, healthy individuals
Ricci et al., 1991 (27)	Pregnant women between 24 and 34 wk gestation	75, with 25 in Mg group (23 completed)	Pilot RCT	MgCl	384 mg (16 mmol)	Variable, until 36 wk or delivery	Side effects were noted in 5 women; diarrhea in 1, fatigue in 1; 2 patients dropped out	Side effects were reported for 20% of the Mg group and 48% of the Ritodrine group. Oral Mg was given after IV Mg sulfate infusion
Spencer et al., 1994 (28)	Adult men (aged 38–75 y)	5	Balance study	MgO plus control diet	Average 576 mg (24 mmol)	28 d	No GI symptoms	Balance study to evaluate calcium and Mg intestinal absorption
Stendig-Lindberg et al., 1993 (29)	Postmenopausal women (mean age, 51.6 ± 10.6 y)	31, with 23 symptom-free controls	Open age-matched control study	Mg(OH)_2_	Dose titration: 250–750 mg/d (10–31 mmol)	6 mo	No side effects due to treatment were observed	No patients withdrew from the study at 6 mo. Mg was taken on an empty stomach at bedtime

Abbreviations: AE, adverse event; CHF, congestive heart failure; GI, gastrointestinal; Mg, magnesium; MgO, magnesium oxide; Mg(OH)_2_, magnesium hydroxide; RCT, randomized controlled trial.

a These studies were cited in the 1997 Institute of Medicine report (1).

The study by Marken et al. [23] is notable because 36% of participants (18 out of 50) reported diarrhea. However, the use of the term *diarrhea* can be questioned because the complaints ranged from mild changes in stool consistency, which could be beneficial, to symptoms distressing enough to cause withdrawal from the study. Only 5 individuals withdrew when taking the 476 mg/d Mg supplement, which is much higher than the current UL and may have been consumed without food. Some subjects did report a lessening of symptoms when taking the supplement with food.

The study by Fine et al. [22] was the only one that defined a priori diarrhea as an outcome objective. This controlled metabolic unit study evaluated the effects of increasing supplemental Mg to determine a dose threshold to induce osmotic diarrhea by monitoring fecal Mg loss. However, this study could be of limited use for setting the UL because participants drank a solution during 3 meals and consumed a snack that provided excessively high total Mg intake of 1160, 2332, or 4690 mg/d for 4 d. Even with these high dosages, diarrhea was induced in only 5 subjects. The authors concluded that the UL of normal for the fecal output of soluble Mg was 355 mg (14.6 mmol)/d and 1098 mg (45.2 mmol)/L fecal concentration. Applying these criteria, they determined that there were only 21 cases in which Mg contributed to diarrhea among the 359 individuals with chronic diarrhea. Fifteen of these individuals were ingesting very high amounts of Mg from sources such as antacids and supplements. The other 5 had other health conditions, including Crohn’s disease and peptic ulcer, that likely confounded the role of Mg in causing diarrhea.

The study by Ricci et al. [27] involved pregnant women in their last trimester who were supplemented with 384 mg Mg/d shortly after receiving tocolytic intravenous Mg sulfate. Of the 25 women receiving the Mg supplement, only 1 reported diarrhea. However, there was no indication that the intake of over-the-counter supplements or medications, which could contain Mg, was determined or limited during Mg supplementation.

The study by Bashir et al. [21] involved individuals with congestive heart failure. These participants had a mean compromised ejection fraction of 25.9% and were taking multiple medications, so these findings are not generalizable to the general healthy population. The 2 individuals that reported diarrhea with intakes of 360 mg/d had a prior history of nonspecific dyspeptic symptoms. In addition, there was no indication that the intake of over-the-counter supplements or medications, which could contain Mg, was determined or limited during the study. Thus, the total intake of nonfood Mg could have been higher than the indicated 360 mg/d for some individuals in this study.

In summary, the current 1997 UL for Mg was based on the presence of diarrhea in a few individuals in a minority of studies and did not, for the most part, represent a comparison of this side effect in oral Mg supplementation compared with placebo studies.

## Recent and needed evidence for re-evaluating the UL for Mg

### Methods

Using the same format and criteria utilized by the IOM DRI Committee [1] to establish the 1997 UL for Mg, an updated PubMed search was performed for intervention studies published between 1997 and 2022 that reported on gastrointestinal adverse events in generally healthy adult populations. Studies that involved patients with acute illness or used intravenous Mg sulfate were excluded. Studies that included diarrhea as an a priori adverse event as an outcome of interest, and Mg as a single-ingredient product, were included in the review. Additional studies were identified using the authors’ Endnote database collection. In addition, the CAERS database was searched from January 2004 until June 30, 2022 (most recent available data) for adverse events from single-ingredient, “suspect” Mg products. This search methodology is presented in Table 2.

**TABLE 2:** FDA CAERS database search: magnesium adverse events

Category	No. of AEs
Vitamin, mineral, protein, or unconventional diet (human/animal category)	79,897
Mg-containing products	310
Suspect AE category products	263
Single-ingredient Mg products	136
Gastrointestinal AEs^a^	40
Diarrhea-specified AEs	15

Abbreviations: AE, adverse event; CAERS, Center for Food Safety and Applied Nutrition Adverse Event Reporting System; Mg, magnesium.

a AEs included nausea, vomiting, diarrhea, or gastrointestinal pain or discomfort.

**FIGURE 1:**
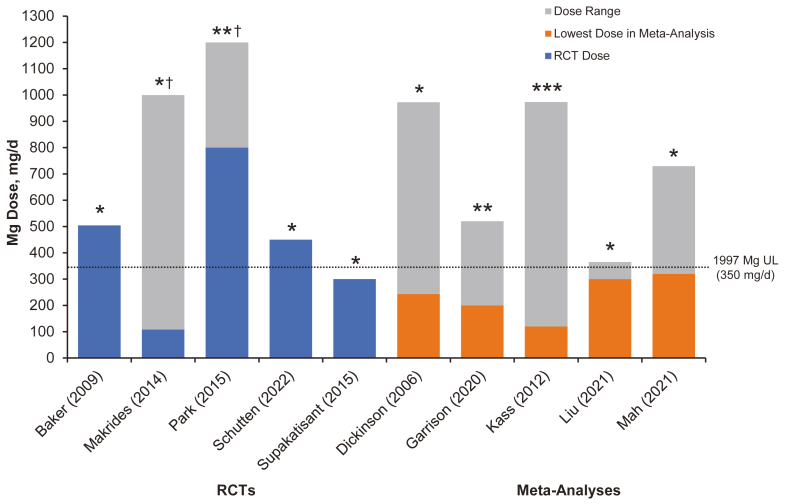
Magnesium dose (range) of oral supplement trials measuring diarrhea side effects compared to the tolerable upper intake level Five RCTs and 5 meta-analyses show the Mg dose range for each study. In the 5 meta-analyses, the lowest dose administered is depicted in orange, and the gray bars represent the full range of doses administered. Only one study [Supakatisant and Vorapong Phupong, 2015 (39)] administered doses below the DRI UL of 350 mg/d. ∗No difference in diarrhea between Mg and control groups. ∗∗More diarrhea in the Mg group than in the control group (not significant). ∗∗∗Inconclusive: 60% of studies reported diarrhea or mild gastrointestinal effects; 40% did not. No statistical analysis was performed. ^†^These RCTs included dose ranges (gray bars) of 128–1200 mg/d [Makrides et al., 2014 (36)] and 800–1200 mg/d [Park et al., 2014 (37)].

No balance or metabolic unit studies evaluating diarrhea as an a priori adverse event were identified. Table 3 and Figure 1 present findings from the studies that were identified; 5 were meta-analyses [[30], [31], [32], [33], [34]], and 5 were randomized controlled trials (RCTs) [[35], [36], [37], [38], [39]]. Seven studies found no significant difference in the occurrence of diarrhea between groups with supplemental Mg intakes of 128–1200 mg/d and control groups [30,[33], [34], [35], [36],^38^,^39^]. Two meta-analyses [31,32] and 1 RCT [37] described differences between groups.

**TABLE 3:** Studies that prespecified evaluation of side effects or adverse event outcomes, 1997–2022

Reference	Population	Study design	Sample size	Mg formulation	Daily dose	Duration	Side effects/AEs	Comments
Baker et al., 2009 (35)	Men and women with elevated blood pressure and implantable cardioverter defibrillator (mean age, 61–68 y)	Double-blind RCT	70	Mg L-lactate	504 mg (21 mmol)	12 wk	Overall incidence of AEs and rate of discontinuations due to AEs were similar in both the Mg L-lactate and placebo groups. The most commonly reported AEs included pill burden, diarrhea, fatigue, itching, and infection	20 patients dropped out by week 12; data analysis was based on 50 patients. 86% of patients enrolled had Mg deficiency. Patients were instructed to take study medication without regard to meals
Dickinson et al., 2006 (30)	Adults with essential hypertension (overall mean age, 54 y; range, 20–77 y)	Meta-analysis of 12 RCTs	545	MgO (3 studies); Mg pidolate and Mg lactate (3 studies); Mg lactate and citrate; Mg aspartate; Mg aspartate hydrochloride (3 studies); Mg (not specified)	Mean: 413 mg (17 mmol); range, 243–972 mg; range, 10–40 mmol	Median follow-up duration of 11 wk (range, 8–26 wk)	Meta-analyses restricted to GI effects and other AEs showed no difference in risk between Mg (risk difference = 0.00 [95% CI, −0.05 to 0.05], I^2^ = 0%) and control groups (risk difference = 0.00 [95% CI, 0.07 to 0.06], I^2^ = 0%)	Withdrawal from treatment for all causes was 7% among participants receiving Mg and 8% among the control group. Patients in 4 studies were taking antihypertensive medication (e.g., calcium antagonists, β-blockers, ACE inhibitors, thiazides, spironolactone, and α-blockers)
Garrison et al., 2020 (31)	Individuals with skeletal muscle cramps for multiple conditions (mean age, 61–69 y)	Meta-analysis of 11 trials	735	Mg lactate and Mg citrate; tri-Mg dictrate; slow-release Mg lactate; Mg bisglycinate; 8% milk of Mg suspension; MgO and Mg aspartate	200–520 mg (8.2–21.4 mmol)	14–56 d	Diarrhea was experienced by 11%–37% of the Mg group and 10%–14% of the control group. Withdrawals due to GI AEs were not significantly different from placebo	1 study enrolled 29 people with liver cirrhosis, and 1 study used IV Mg sulfate; 3 studies used once-daily dosing
Kass et al., 2012 (32)	Hypertensive and normotensive men and women from 12 different countries (mean age, 50–52 y)	Meta-analysis of 22 RCTs	1173	7 different Mg formulations: oxide, aspartate, chloride, lactate, citrate, pidolate, and hydroxide	Mean: 410 mg (16.9 mmol); (range, 120–973 mg; range, 5–40 mmol]	Mean, 11.3 wk (range, 3–24 wk)	13 studies reported AEs from the Mg and placebo treatments. AEs were largely either diarrhea or nonspecific mild abdominal or bone pain. Only 3 studies reported serious AEs that led to withdrawal (treatment arm not specified in 2 studies)	Some patients were taking antihypertensive medications and diuretics. Greater BP lowering would have been seen at dosages >370 mg/d
Liu et al., 2021 (33)	Pregnant women with leg cramps (mean age, 19–45 y)	Meta-analysis of 4 RCTs	332	Mg lactate or citrate (2 studies), Mg bisglycinate chelate (1 study), and Mg citrate (1 study)	300–360 mg (12.3–14.8 mmol)	2–4 wk	No significant side effects in the treatment group compared with the control group (OR, 1.82 [95% CI, 0.90 to 3.69]; *P* = 0.094)	Documentation of side effects was part of the inclusion criteria in the meta-analysis. Doses given 2 times/d (2 studies), 3 times/d (1 study), and once daily (1 study)
Mah and Pitre, 2021 (34)	Older adults with insomnia, mostly without comorbidities (mean age, 51–80 y)	Meta-analysis of 3 RCTs	151	MgO or citrate	320–729 mg (13.1–30.0 mmol)	20 d to 8 wk	Participants in 1 study reported soft stools	Doses are given 2 or 3 times/d
Makrides et al., 2014 (36)	Morbidity and mortality outcomes for pregnant women and their infants from 7 different countries	10 RCTs	9090	MgO, 1000 mg (41 mmol) (1 trial); Mg citrate, 365 mg (15 mmol) (1 trial), and 340 mg 14 mmol) from 9 to 27 wk gestation (1 trial); Mg gluconate, 108–161 mg (4.4–6.6 mmol) (1 trial) and 215 mg (8.8 mmol) (1 trial); Mg aspartate, 15 mmol (365 mg) (3 trials), Mg aspartate hydrochloride 365 mg (1 trial); and Mg stearate, 128 mg (5.3 mmol) (1 trial)			4 trials (1388 women) reported on GI symptoms and found no significant difference between the Mg group and control group (RR, 0.88 [95% CI, 0.69 to 1.12])	Compositions of the Mg supplements, gestational ages at commencement, and doses administered varied
Park et al., 2014 (37)	Postmenopausal women with a history of hot flashes (84% ≥ 50 y)	Double-blind RCT with 4 study arms (2 Mg and 2 placebo)	289	MgO	800 or 1200 mg (32.9–49.4 mmol)	8 wk	The incidence of diarrhea with Mg was more prevalent than in the placebo arm; constipation was reported less frequently with Mg. There were no significant toxicity differences between the study arms	Toxicities were included as a secondary study end point and coded using CTCAE version 4. A self-reported validated survey instrument and telephone interviews were used to collect data on the frequency and severity of hot flashes and potential toxicities
Schutten et al., 2022 (38)	Adults with overweight or slight obesity (mean age, 63 y)	Double-blind, parallel-group RCT	164	Mg citrate, MgO, or Mg sulfate.	All are equivalent to 450 mg	24 wk	6 patients discontinued the study due to GI symptoms (2 from Mg citrate, 2 from Mg sulfate, and 2 from placebo). Mild diarrhea was noted for 5 patients taking Mg citrate, 1 taking Mg sulfate, 1 taking MgO, and 2 taking placebo	Mg supplement is taken 3 times/d. Patients completed the PHQ-15 (which includes 3 GI questions) and a 3-d food diary at baseline and study end; 15% were taking antihypertensive medications
Supakatisant and Vorapong Phupong, 2015 (39)	Pregnant women 14–34 wk of gestation with leg cramps (mean age, 29 y)	Double-blind RCT	86	Mg bisglycinate chelate	300 mg (12.3 mmol)	4 wk	No significant differences between groups in terms of side effects such as nausea (*P* < 0.10) and diarrhea (*P* < 0.27)	Compliance evaluated from returned tablets showed no differences between groups (*P* = 0.26)

Abbreviations: AE, adverse event; CTCAE, Common Terminology Criteria for Adverse Events; GI, gastrointestinal; IV, intravenous; Mg, magnesium; MgO, magnesium oxide; PHQ-15, Patient Health Questionnaire-15; RCT, randomized controlled trial; RR, risk ratio.

The studies that indicated some differences between Mg-supplemented participants and controls were not supportive of a UL as low as 350 mg/d. In a meta-analysis of 11 trials in adults with skeletal muscle cramps from multiple conditions, Garrison et al. [31] found some important differences in gastrointestinal disturbances between groups given placebo versus a Mg supplement of 520 mg/d. However, withdrawals because of gastrointestinal adverse events were not significantly different between the Mg-supplemented and placebo groups, as noted in only 4 out of 11 trials that documented withdrawals.

The meta-analysis of 22 studies by Kass et al. [32] in hypertensive and normotensive men and women also had groups supplemented with Mg much higher than 350 mg/d. Only 3 of 13 studies that reported adverse events noted if diarrhea was an adverse event that led to withdrawal from the study, and the treatment arm was not specified in 2 of these studies. The earlier meta-analysis by Dickinson et al. [30] in adults with essential hypertension included only 12 RCTs, 10 of which were also included in the later meta-analysis by Kass [32]. Only 4 studies in the Dickinson et al. meta-analysis included data on withdrawals where the intervention arm was identified with Mg as the causative agent in 2 of the trials, similar to the findings of Kass et al. [32]. These meta-analyses enrolling 2936 individuals demonstrate a low rate of mild side effects and support the use of Mg doses >350 mg/d.

Of the 5 RCTs, the study by Schutten et al. [38] in adults with overweight or slight obesity documented a slight difference between groups. In that study, the Mg supplements were equivalent to 450 mg/d. Only 6 participants out of 164 (ie, <4%) discontinued the study because of gastrointestinal symptoms; 2 of 48 were supplemented with Mg citrate, 2 of 48 were supplemented with Mg sulfate, and 2 of 25 were given placebo. Imprecisely described mild diarrhea, which could have included just stool softening, occurred in 5 of 48 participants supplemented with Mg citrate, 1 of 48 in each supplemented with Mg sulfate and Mg oxide, and 2 given placebo. The similarity in the percentage of gastrointestinal complaints between the placebo and treatment groups suggests that other factors besides Mg contributed to the complaints. The RCT by Park et al. [37] noted a higher prevalence of diarrhea in the Mg group at both the 800 and 1200 mg doses. Still, the authors noted no significant toxicity differences between groups. Collectively, the RCTs enrolling 9699 adults with varying chronic conditions, hypertension, pregnancy outcomes for mother and infant, postmenopausal women with hot flashes, and overweight or slightly obese individuals also demonstrate a low rate of mild side effects and support the use of Mg doses >350 mg/d.

Lastly, findings from the CAERS database found that only 0.0005% of gastrointestinal adverse events were attributed to an Mg supplement. The findings reported in TABLE 1, TABLE 2, TABLE 3 show that setting a UL for Mg as a supplement is problematic due to small sample sizes, lack of high-quality data, and higher heterogeneity in many of the meta-analyses. They also indicate that a UL of 350 mg/d is most likely low. Individual gastrointestinal symptoms in response to Mg supplementation vary greatly and depend on the Mg formulation used, the duration of supplementation, and differences in diet and health status. Mg supplements are best taken with food [40]. Large doses should be spread throughout the day to avoid exceeding the absorption threshold for Mg, which is 30%–40%, with normal dietary intakes [41]. For healthy individuals, the efficiency of the kidney in eliminating Mg is great enough that even excessive ingestion rarely leads to significant increases in serum Mg concentrations [42].

## Diarrhea as an adverse event outcome measure for the UL

Diarrhea is a common complaint. It has been estimated that about 179 million cases of acute diarrhea occur in the United States each year [43]. The National Institute of Diabetes and Digestive and Kidney Diseases (NIDDK) defines diarrhea as loose, watery stools occurring ≥3 times/d [43]. Diarrhea may be acute (1–2 d), persistent (>2 and <4 wk), or chronic (≥4 wk). Acute diarrhea is more common than persistent or chronic diarrhea [43].

Most of the studies in TABLE 1, TABLE 3 do not indicate the criteria the investigators used to identify diarrhea or whether the criteria conformed to the NIDDK medical definition and duration. If diarrhea was identified as an adverse event, it was typically self-reported and not highly characterized. Future clinical studies involving diarrhea as an outcome of oral Mg supplementation should institute a standardized format for its data collection. One possible format is the Bristol Stool Form Scale [44], a frequently used measure in gastroenterology practice and research. Stools are categorized into 1 of 7 stool types ranging from type 1 (hard lumps) to type 7 (watery diarrhea). Future clinical trials on diarrhea attributable to Mg supplement also should follow a format such as that described recently by Ashmead et al. (abstract only) [45]. The investigators used a randomized, double-blind, three-cohort crossover design to evaluate the gastrointestinal tolerability of 3 different forms of supplemental Mg at 300, 400, and 650 mg/d compared with a placebo. Gastrointestinal symptoms were generally mild, with no significant differences between treatments and placebo. Ashmead et al. [45] concluded that Mg supplementation resulted in significant improvements in bowel habits, including easier stool passage.

## Factors affecting the response to Mg salts

Except for osmotic diarrhea, there is no evidence of harmful effects caused by high Mg intakes from supplements [1]. Also, reasonably high oral Mg supplementation, if properly done, can be achieved without causing diarrhea. Choosing the right salt form should be among the items to consider for Mg supplementation. Mg supplements are available in a variety of salt forms, including Mg oxide, Mg citrate, Mg chloride, Mg gluconate, Mg malate, and Mg glycinate [46]. Mg gluconate and Mg chloride have been preferred for oral replacement because they cause diarrhea less often than other salt forms [47]. Mg carbonate has been avoided as a supplement because it apparently is not soluble enough to be absorbed in desired amounts. Some of the studies in Table 3 suggest that some forms of Mg may be more prone to induce stool softening, diarrhea, and/or gastrointestinal complaints.

Other factors affecting the response to Mg salts include their solubility. For example, Mg citrate, which might affect stools more than Mg oxide, is highly soluble in water, whereas Mg oxide is poorly soluble even in acid solution. Mg salts in effervescent tablets have a higher solubility than single-dose tablets [48]. As indicated above, taking a high amount of Mg in divided doses instead of in a single dose and taking supplements with food can mitigate the chances of gastrointestinal symptoms [40]. The variability in the bioavailability of Mg preparations are important features that should be taken into account when setting a UL for Mg supplements.

## Conclusion

The prevalence of Mg undernutrition, the current literature and supplementation practices described above, and the very low number of CAERS reports attributable to Mg supplements strongly support the suggestion that the UL for Mg supplements is too low and needs to be re-evaluated. This evaluation should also include directions for the appropriate use of Mg supplements. Design of RCTs with diarrhea as an a priori outcome measure determined by definitive criteria for its incidence and duration would facilitate the setting of an appropriate UL for Mg supplements. The greater goal of increasing the UL for Mg supplements would be to decrease the number of Americans with intakes below the EAR and potentially reduce the risk of a number of chronic diseases.

## Acknowledgments

The authors’ responsibilities were as follows—RC designed research; RC, AR, and FN conducted research; RC and AR, and FN analyzed data; and RC, AR, FN, and CW wrote the paper. RC had primary responsibility for the final content. All authors read and approved the final manuscript.

### Author disclosures

AR holds a patent on a magnesium cream product. All other authors report no conflicts of interest.

### Funding

This work was self-funded by CMER (The Center for Magnesium Education and Research). No authors received payment for their work.
